# Higher NADH Dehydrogenase [Ubiquinone] Iron–Sulfur Protein 8 (NDUFS8) Serum Levels Correlate with Better Insulin Sensitivity in Type 1 Diabetes

**DOI:** 10.3390/cimb44090266

**Published:** 2022-08-26

**Authors:** Justyna Flotyńska, Daria Klause, Michał Kulecki, Aleksandra Cieluch, Regina Chomicka-Pawlak, Dorota Zozulińska-Ziółkiewicz, Aleksandra Uruska

**Affiliations:** 1 Department of Internal Medicine and Diabetology, Poznan University of Medical Sciences, Raszeja Hospital, Mickiewicza 2, 60-834 Poznan, Poland; 2 Doctoral School, Poznan University of Medical Sciences, Collegium Stomatologicum, Bukowska 70, 60-812 Poznan, Poland; 3 Department of Hypertensiology, Angiology and Internal Medicine, Poznan University of Medical Sciences, University Hospital of Lord’s Transfiguration, Długa ½, 61-848 Poznan, Poland

**Keywords:** diabetes mellitus, mitochondria, insulin resistance

## Abstract

Objective: The aim of the study was to evaluate NADH dehydrogenase [ubiquinone] iron–sulfur protein 8 (NDUFS8) serum concentration as a marker of Complex I, and the relationship with insulin resistance in type 1 diabetes mellitus (T1DM). Design and methods: Participants were adults with T1DM, recruited over the course of 1 year (2018–2019). NDUFS8 protein serum concentration was measured using the ELISA test. Insulin resistance was evaluated with indirect marker estimated glucose disposal rate (eGDR). The group was divided on the base of median value of eGDR (higher eGDR—better insulin sensitivity). Results: The study group consists of 12 women and 24 men. Medians of eGDR and NDUFS8 protein concentration are 7.6 (5.58–8.99) mg/kg/min and 2.25 (0.72–3.81) ng/mL, respectively. The group with higher insulin sensitivity has higher NDUFS8 protein serum concentration, lower waist to hip ratio (WHR), body mass index (BMI), and they are younger. A negative correlation is observed between NDUFS8 protein serum concentration and WHR (rs = −0.35, *p* = 0.03), whereas a positive correlation is observed between NDUFS8 protein serum concentration and eGDR (rs = 0.43, *p* = 0.008). Univariate logistic regression shows a significant association between insulin sensitivity and lower age, as well as a higher NDUFS8 serum level. A multivariate logistic regression model confirms the significance (AOR 2.38 (1.04–5.48). *p* = 0.042). Multivariate linear regression confirms a significant association between insulin sensitivity and better mitochondrial function (beta = 0.54, *p* = 0.003), independent of age, duration of diabetes, and smoking. Conclusions: Higher NDUFS8 protein serum concentration is associated with higher insulin sensitivity among adults with T1DM.

## 1. Introduction

Type 1 diabetes mellitus (T1DM) is a disorder characterized by the destruction of pancreatic β cells. This process leads to complete insulin deficiency [1,2]. The incidence of DM is continually increasing all over the world, as well as in the Polish population [3,4,5]. Adults with T1DM experience a lower health-related quality of life, are more frequently unemployed, and have more sick leave per year in contrast to the general population [6]. These facts are attributed to the development of chronic complications: neurovascular and macrovascular [7]. The development of chronic complications is connected with several risk factors, most prominently insulin resistance (IR) [8,9] and hyperglycemia [10].

Insulin resistance is mostly connected to obesity and diabetes mellitus type 2. However, more and more young people diagnosed with T1DM are overweight or obese at the moment of diagnosis [11,12]. Moreover, IR also develops during T1DM as a result of exogenous insulin treatment and aging. Insulin therapy causes abdominal obesity, as do smoking and physical laziness. A widely used, indirect, validated marker of IR in DM1 is estimated glucose disposal rate (eGDR), which was created well with IR measured by hyperinsulinemic–euglycemic clamps, the gold diagnostic standard of IR in DM1 [13]. eGDR was proposed as a new practical measure of IR. An advantage of eGDR is the simplicity. Moreover, eGDR has a prognostic significance, as studies show that low eGDR is associated with an increased [9] risk of vascular complications, as well as mortality, in T1DM [14].

The main pathomechanisms of hyperglycemia-based complications, other than the enhanced glycolysis that is the main pathway of glucose metabolism, are increased flux through the polyol pathway, intracellular production of advanced glycation end products (AGE) precursors, protein kinase C (PKC) isoforms activation, and increased hexosamine pathway activity. Furthermore, it is also known that IR is strongly associated with micro and macrovascular complications [8,9,15,16]. All these mechanisms have a common underlying process: increased reactive oxygen species (ROS, free radicals) production [17].

Oxidative stress is defined as a disturbance in the balance between the production of ROS and antioxidant defenses, which leads to cellular damage [18]. The main organelle responsible for ROS production is the mitochondrion. In the inner membrane of this organelle exist four complexes, creating the mitochondrial respiratory chain. This whole structure is responsible for electron transport. During each one of these four steps, partially reduced oxygen intermediates are generated. Some of them are very stable and can be stored by the enzyme cytochrome c oxidase until all the electrons are transferred. However, 1–2% of the oxygen molecules are converted to superoxide anion radicals. It happens mainly in Complex I (mitochondrial NADH: ubiquinone oxidoreductase) and Complex III (ubisemiquinone) [19,20,21].

In hyperglycemic conditions, in cells there is increased glucose oxidation during the tricarboxylic acid (TCA) cycle, caused by persistent hyperglycemia and enhanced B-oxidation of free fatty acids (in macrovascular endothelial cells) due to insulin resistance. This leads to a situation where more electron donors, mainly NADH, are released and more protons pass through the mitochondrial membrane, thus, a higher voltage is created [22]. As a result, more ROS are produced and released. The redox balance in DM between NADH and NAD^+^ is highly elevated [23,24], leading to enhanced ROS production, which shows the insufficiency in mitochondrial NADH dehydrogenase activity.

It is proven that ROS production is due to high proton potential [25]. Some trials were conducted with patients with T2DM, in which they discovered the possible role of mitochondrial Complex I impairment [15]. Complex I is also proposed as a responsible source of oxidative stress among patients with polycystic ovarian syndrome with IR [26]. We found that NADH dehydrogenase [ubiquinone] iron–sulfur protein 8 (NDUFS8 protein) can be measured in human serum. It is a subunit of NADH dehydrogenase (ubiquinone), also known as Complex I, encoded by the NDUFS8 gene [27]. The NDUFS8 gene is located on chromosome 11q13. It spans about 6 kb, and contains seven exons ranging in size from 51 to 186 bp. Expression of the gene is ubiquitous, but is predominant in the heart and skeletal muscles [28]. Mutations in this gene are associated with Leigh syndrome (neurodegenerative disorder) [29]. No studies have been conducted with patients with T1DM. 

The aim of the study was to evaluate the presence of Complex I in serum by measuring NADH dehydrogenase [ubiquinone] iron–sulfur protein 8 serum level, and the relationship with insulin resistance in type 1 diabetes. We suggest that NDUFS8 serum concentration probably reflects the turnover of mitochondria.

## 2. Methodology

### 2.1. Study Design

The study design: a cross-sectional, single-center study. The study group consisted of 36 adults with T1DM. Participants were recruited over the course of 1 year (2018–2019). The inclusion criteria were: age above 18 years old, and at least five years history of T1DM confirmed in the past with T1DM antibodies (to glutamic acid decarboxylase—antiGAD; islet cells—ICA; islet tyrosine phosphatase 2—IA2), whereas the exclusion criteria were: CRP > 5 mg/L, unstable hypo/hyperthyroidism (TSH beyond normal range), other endocrinological disorders, contagious diseases, renal or liver diseases, pregnancy, antineoplastic therapy in less than 2 years, and any psychological or psychiatric disorder. Subjects were informed about the aim of the study and signed a consent form. The study was approved by the local Ethical Committee at the Medical University of Poznan (resolution no. 15/18). We confirm that all methods were performed in accordance with the relevant guidelines and regulations. Everyone was treated with functional intensive insulin therapy at the onset of diabetes. This method aims to mimic physiology, and two types of insulin are used: long-acting insulin analogues (so-called basal insulin) and rapid-acting analogues (used before main meals) with insulin pen devices or rapid acting analogues in the insulin therapy with personal insulin pumps. All the participants completed a standardized questionnaire including details of age, sex, chronic diseases, medicines, family history regarding diabetes and pack–years of cigarette smoking, duration of diabetes, blood glucose self-control, and medical history. Participants underwent a complete physical examination with anthropometric measurements (weight, height, waist and hip circumference) and blood pressure check.

### 2.2. Insulin Resistance Markers

Insulin resistance was evaluated with indirect markers (the estimated glucose disposal rate—eGDR, waist to hip ratio—WHR, body mass index—BMI, TG/HDL—cholesterol). The clinical characteristics of the whole study group, and according to the presence of insulin resistance, are shown in Table 1. The estimated glucose disposal rate (eGDR) was calculated by the following mathematical formula: 24.31–12.22 (WHR)—3.29 (hypertension 0/1)—0.57 (HbA1c) [mg/kg/min] [30]; waist to hip ratio (WHR) was checked using the non-elastic measuring tape with the resolution of 0.5 cm and calculated from the following equation: WHR = waist circumference [cm]/hip circumference [cm]. It was assumed that the higher the eGDR, the higher tissue sensitivity for insulin.

### 2.3. Blood Tests

Blood samples were collected in a fasting state using the S-Monovette blood collection system. We assessed glycated hemoglobin level (A1c), serum total cholesterol, high-density lipoproteins (HDL) cholesterol, low-density lipoproteins (LDL) cholesterol, and triglycerides (TG).

### 2.4. NADH Dehydrogenase [Ubiquinone] Iron–Sulfur Protein 8 Measurement

NADH dehydrogenase [ubiquinone] iron–sulfur protein 8 (NDUFS8 protein), mitochondrial (also known as NADH–ubiquinone oxidoreductase 23 kDa subunit, Complex I-23kD (CI-23kD), or TYKY subunit) serum concentration was measured using the ELISA test (EIAAB SCIENCE INC, WUHAN 3F, Building A4, Biopark, Optics Valley, Wuhan 430079, China). This immunoassay kit allows for the in vitro quantitative determination of human NADH dehydrogenase [ubiquinone] iron–sulfur protein 8, mitochondrial, its concentrations in serum, plasma, urine, tissue homogenates and cell culture supernates, and other biological fluids. The NDUFS8 protein is a subunit of NADH dehydrogenase (ubiquinone) also known as Complex I, which is located in the mitochondrial inner membrane and is the largest of the five complexes of the electron transport chain. The reference range for ELISA kit is 0.312–20 ng/mL, sensitivity 0.1 ng/mL.

### 2.5. Statistical Analysis

The statistical analysis was performed using the STATISTICA software v 13.3 (TIBCO Software Inc., Hillview Avenue Palo Alto, USA). The normality of distributions was tested using Kolmogorov–Smirnov’s test with Lilliefors correction. Due to lack of normality, non-parametrical tests were performed. All data are expressed as medians and interquartile range (IQR) or percentage of subjects. Usually, the value of the cut-off point for eGDR is 7.5 mg/kg/min, according to the clamp technique of DeFronzo [30]. In this case, the patients were divided into two groups, below and above eGDR median, because a higher eGDR value shows insulin sensitivity better (less IR). The Mann–Whitney U and chi^2^ tests were used to assess differences between groups. The multivariate linear regression, R Spearman correlation was performed. Differences with a probability value < 0.05 were considered statistically significant.

## 3. Results

The study group consists of 36 adults with T1DM, 12 women (33%) and 24 men (67%), aged 39.5 (28.0–46.5) years, with the duration of the disease lasting 22 (15–26) years and HbA1c 8.35 (6.92–9.78)% (Table 1). Medians of chosen insulin resistance indicators among the investigated group are: eGDR 7.6 (5.58–8.99) mg/kg/min and WHR 0.88 (0.83–0.92). The median of the NDUFS8 protein concentration is 2.25 (0.72–3.81) ng/mL (Table 1). People with eGDR above the median (less IR) are proven to have higher NDUFS8 protein serum concentration and TG/HDL-c ratio, lower WHR and BMI, and they are younger (Table 1).

**Figure 1:**
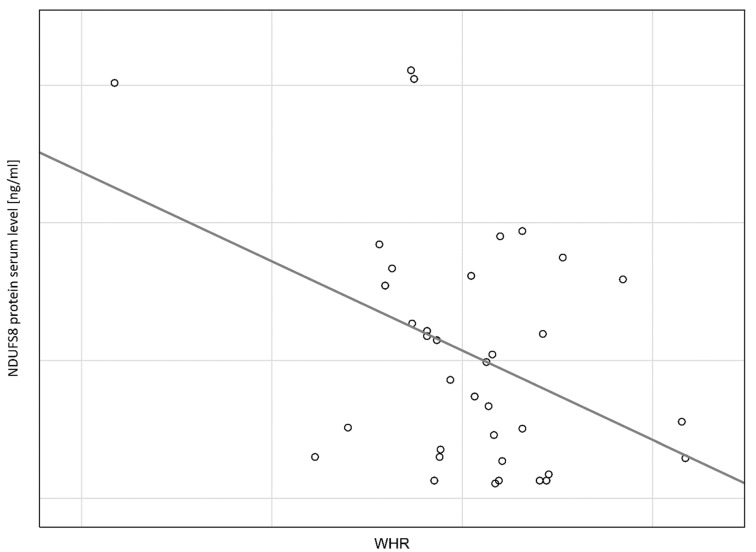
Negative relationship between NDUFS8 protein serum concentration and WHR. (rs = −0.35, *p* = 0.03).

**Figure 2:**
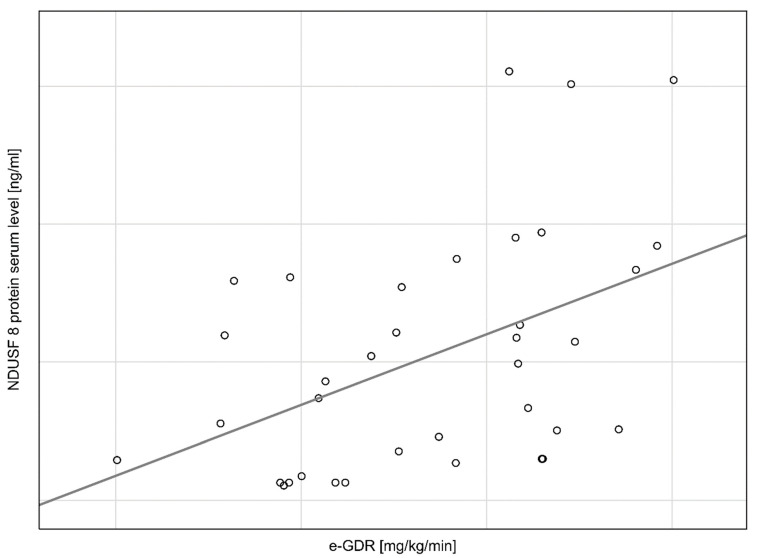
Positive relationship between NDUFS8 protein serum concentration and eGDR. (rs = 0.43, *p* = 0.008).

The negative correlation is observed between NDUFS8 protein serum concentration and WHR (rs = −0.35, *p* = 0.03) (Figure 1), whereas positive correlation is observed between NDUFS8 protein serum concentration and eGDR (rs = 0.43, *p* = 0.008) (Figure 2). We do not observe a statistically significant correlation between NDUFS8 protein serum concentration and TG/HDL-c ratio (rs = −0.12, *p* = 0.51) or BMI (rs = −0.04, *p* = 0.83).

Univariate logistic regression shows a significant association between insulin sensitivity and lower age, as well as a higher NDUFS8 serum level. A multivariate logistic regression model confirms the significance. This correspondence is independent of diabetes duration and pack–years (adjusted odds ratios = 0.81 (0.67–0.97); *p* = 0.019, and 2.38 (1.04–5.48); *p* = 0.042, respectively) (Table 2). The higher the serum level of NDUFS8, the greater the chance of a higher level of eGDR (better insulin sensitivity).

Multivariate linear regression confirms a significant association between insulin sensitivity and a higher concentration of NDUFS8 in serum (beta = 0.54, *p* = 0.003), independent of age, duration of diabetes, and smoking (Table 3).

## 4. Discussion

The study was designed to assess the mitochondrial function in adults with T1DM, and its relationship with insulin resistance (IR). We found that people with T1DM who have better insulin sensitivity (eGDR above the median) have higher NADH dehydrogenase [ubiquinone] iron–sulfur protein 8 (NDUFS8) serum concentration. The relationship is independent of other important factors. There is still lack of information on the exact mechanisms of how Complex I function is indeed impaired, whether it is increased or decreased in activity among patients with IR neither, and what is the best way to check it. The relationship is probably two-sided. There is also no reference range of NDUFS8 protein serum concentration in healthy people, nor in people with T1DM. NDUFS8 protein concentration was measured in serum as a simple, non-invasive way that reflects the number and possibly function of mitochondria. As a probable marker of mitochondrial function, it can help us to find patients more prone to the development of IR and, hence, start the prevention strategies accurately and earlier.

Mitochondria are responsible for production of respiratory ATP, and are essential for eukaryotic life. The mitochondria participate in critical central metabolic pathways and they regulate diverse cellular functions. Mitochondrial defects diminishes tissue and organ functional performance, and is commonly considered a feature of the aging process [31]. Dysfunction of mitochondria can result in cell apoptosis/death, metabolic syndrome, diabetes, autoimmune diseases, and cancers [32]. Nowadays, more and more publications describe the circulating mitochondria in human and animal blood. The horizontal transfer of mitochondria between surrounding cells through the formation of tunneling nanotubes, cell fusion, GAP junctions, and microvesicles is possible [33]. In the case of pathological conditions, mitochondria could be released by the activated monocytes [34], diseased organs [35], and oxidative-stressed mesenchymal stem cells [36]. Zahra et al. evaluated the metabolic activity of mitochondria by measuring oxygen consumption rate (OCR) with the Seahorse XF extracellular flux analyzer technology (Agilent). The results show that when isolated, the pellets consume oxygen and are sensitive to Complex I inhibition, suggesting that the extracellular mitochondria remain competent for respiration [37]. On the other hand, Stier said they are unlikely to be fully functional (i.e., capable of oxidative phosphorylation). However, circulating acellular mitochondria may play important physiological roles that remain to be clarified [38]. Zahra et al. suggest that circulating, cell-free, intact mitochondria have crucial biological and physiological roles as systemic messengers in cell–cell communication, by transferring hereditary and nonhereditary constituents [37]. Mitochondria were recently discovered to translocate from one cell to the other [33]. Transfer between differentiated and mesenchymal stem cells was observed as a means to rescue injured tissues [39]. Platelet-derived mitochondria migrate to pancreatic islets through transwell membranes, and enhance human islet β-cell functions [40]. Ongoing studies reveal a novel function of mitochondria directly contributing to cellular reprogramming [41]. Circulating mitochondria can act as a mediator, leading to energy balance and conversation between cells, tissues, and organs. When relocating, presumably, mitochondria are degraded in plasma, and their content is released in the blood stream [42,43], thus, this explains the direct presence of NDUFS 8 in the serum. It is possible that the concentration of NDUFS 8 indicates the amount of mitochondria in the serum and their turnover.

**Figure 3:**
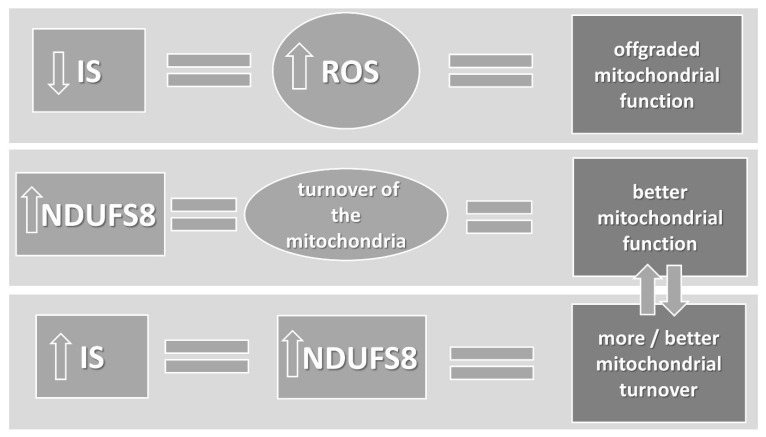
The relationship between NADH dehydrogenase [ubiquinone] iron–sulfur protein 8 (NDUFS 8) and insulin sensitivity (IS).

The main point of improving insulin sensitivity is endurance training, and the related activation of AMP-activated protein kinase (AMPK). AMPK increases ATP generation and decreases ATP consumption [44]. When cellular energy is low, AMPK is activated and influences physiological processes, resulting in increased energy production [45]. Catabolic pathways, including glucose and fatty acid uptake, glycolysis, and fatty acid oxidation are activated by AMPK [46]. Another important process activated by AMPK is mitochondrial biogenesis, and a specific form of autophagy—mitophagy [47]. Mitochondria are the primary production site of reactive oxygen species in cells. By recycling damaged mitochondria, mitophagy is important in the ability to produce ATP, as is the production of new mitochondria [44]. Mitochondrial turnover and migration is an equally important factor that likely influences their function. We do not have data on the relationship between the activity of Complex I subunits and the function of mitochondria, but the interruption of the activity of Complex I, either by toxins or due to genetic disorders such as Leigh’s Syndrome or Leber hereditary optic neuropathy, has debilitating consequences [48]. Petrosillo G. et al., in studies on rats, confirm a strong positive correlation between a decreased Complex I functionality and an increase in ROS production [49]. We, therefore, assume that a higher concentration of single subunits of Complex I determines more mitochondria in human serum and, therefore, their better function (Figure 3).

Zhao et al. identified multipotent stem cells from human cord blood (CB-SC) [50] that phenotypically exhibit embryonic transcription factors. Based on the immune modulation features [51], they develop a “stem cell educator (SCE) therapy” that uses allogeneic CB-SC to “educate” a patient’s immune cells, affecting the progression of autoimmunity in subjects with type 1 diabetes (T1D) [51] and other autoimmune diseases [52]. SCE therapy provides lasting improvement of pancreatic islet β-cell function and C-peptide levels [51]. While exploring the clinical mechanisms underlying the long-lasting therapeutic effects of SCE therapy [40] in ex vivo studies, they found that mitochondria released from platelets can migrate to pancreatic islets, and be taken up by human islet β cells, leading to the improvement of islet β-cell function and C-peptide production [40], supporting the clinical outcomes of the improved health status in patients with type 1 [51,53] and type 2 diabetes [54].

Oxidative stress is the process known to cause IR and, in general, may be involved in T1DM pathogenesis [55]. Oxidative stress induces insulin resistance, and arises from chronic low-grade inflammation due to increased amounts of free fatty acids (FFA) and pro-inflammatory cytokines released from adipose tissue [56,57]. Also, there is an increased flux of FFA from adipocytes, which then undergo B-oxidation. It is exactly the same mechanism of ROS production as hyperglycemia causes. ROS causes mitochondrial DNA mutations, which result in an accumulation of errors in the replication, and ineffective damage repair. Disturbances in oxidative phosphorylation decrease adenosine triphosphate (ATP) production and increase ROS production, leading together to cell function disturbance. Finally, mitoptosis (also known as auto-destruction of mitochondria) occurs [58]. We can assume that the mitochondria, by producing an excessive amount of ROS, destroy themselves. Moreover, it is also proven that the antioxidant levels in DM patients are decreased [59]. There are also studies that indicate that glucose-induced ROS overproduction can lead to IR in skeletal muscle in the process of mitochondrial fusion [60]. All of this constitutes late micro- and macro-vascular complications [17]. The conclusion is: by restricting the mitochondrial ROS production, we can prevent the development of IR, but also act later in the pathomechanism of IR- and hyperglycemia-based complications development.

Victor M. V. et al. report that there can be an association between impaired mitochondrial oxidative metabolism and IR, which plays a huge role in pathogenesis of polycystic ovary syndrome (PCOS), as well as in T2DM and T1DM. The study demonstrates a reduction in NADH oxidation in leukocytes, which indicates that Complex I is the place that is mostly affected by IR [26]. A similar study was conducted by Hernandez-Mijares A. et al., who also checked the rates of oxidative stress and mitochondrial impairment among people with T2DM. The results prove the same conclusion—impaired mitochondrial oxidative metabolism that takes place at Complex I is present among DM2, in comparison with healthy counterparts [25].

There are studies that tried to find out which exactly part of the electron transport chain is responsible for the augmented ROS production that drives the described spiral of unwanted events in T1DM patients’ cells. A T1DM mice model shows that mitochondria with single nucleotide polymorphism in the mitochondrial gene encoding NADH dehydrogenase subunit two (mt-ND2) are characterized by lower reactive oxygen species production, and are more resistant to nitric oxide [61]. Another study shows that subjects with three mtDNA restriction fragment length polymorphism (RFLPs) (morphs) (two are in subunit five of the NADH dehydrogenase gene, and one in the tRNA for threonine), are characterized a higher maximal oxygen uptake (VO2max) in the untrained state than non-carriers. VO2max is a marker associated with insulin sensitivity. Studies carried out in healthy people suggest that certain mtDNA variants may contribute to increasing in VO2max and its response to training [62].

We assume that the higher concentration of the NDUFS8 protein in the serum indicates a significantly greater number of mitochondria in the human body and, therefore, better mitochondrial turnover, which may be result in better mitochondrial function. In this case, the results show a relationship between higher Complex I subunit concentration and better insulin sensitivity. The obtained results seem to confirm the hypothesis of better turnover of mitochondria, however, it must be confirmed in experimental and clinical studies. Therefore, questions arise: is it possible to influence the number of mitochondria, their turnover, and improve mitochondrial function by reducing the IR, or does the number of mitochondria have a positive effect on insulin sensitivity?

The study has several limitations. First of all, the number of patients is small. Furthermore, we measure only the NDUFS8 protein serum level using the ELISA test, excluding other methods of evaluating NADH dehydrogenase activity, and also other mitochondrial enzymes. However, there are no studies in the literature that check the NDUFS8 protein serum level among people with T1DM, which makes this study unique. Also, we have not assessed insulin resistance using the DeFronzo clamp technique [22], which is the gold diagnostic standard. Our results provide the basis for future research, assessing function of mitochondria, its relationship with IR, and the factors that can influence both mitochondrial function and IR. All of this helps in slowing down, or even stopping, the development of chronic complications, and improving the prognosis of T1DM.

## 5. Conclusions

A higher NDUFS8 protein serum concentration is associated with higher insulin sensitivity among people with T1DM, and might reflect better mitochondrial turnover. The results in this study confirm the importance of insulin sensitivity as a factor that improves mitochondrial migration.

## Figures and Tables

**Table 1:** Comparison of whole subjects, group above and below glucose disposal rate(eGDR). Median of eGDR is 7.6 mg/kg/min. Data presented as median (IQR)/n(%).

	All Subjects n = 36	eGDR above Median n = 16	eGDR below Median n = 20	*p*
Sex males n (%)	24 (67)	10 (63)	14 (70)	0.640
Smokers n (%)	13 (36)	6 (38)	7 (35)	0.878
Pack-years [years]	0.0 (0.0–2.4)	0.0 (0.0–1.25)	0.0 (0.0–4.0)	0.690
Age [years]	39.5 (28.0–46.5)	29.0 (24.5–35.0)	43.0 (39.5–48.5)	0.003
Diabetes duration [years]	22.0 (15.0–26.0)	18.5 (12.5–23.5)	25.0 (20.0–27.5)	0.039
Weight [kg]	74.6 (64.8–84.8)	66.5 (61.5–74.6)	78.5 (69.1–88.8)	0.063
BMI [kg/m^2^]	24.3 (22.4–27.1)	23.0 (22.2–24.3)	25.9 (23.4–27.3)	0.025
Waist circumference [cm]	0.88 (0.83–0.92)	82.0 (76.0–91.5)	91.0 (85.5–105.5)	0.003
Systolic blood pressure [mmHg]	122 (113–130)	122 (113–127)	125 (114–134)	0.278
Diastolic blood pressure [mmHg]	80 (73–85)	80 (70–85)	80 (74–86)	0.405
A1C [%]	8.3 (7.3–9.1)	8.4 (7.3–9.3)	8.2 (7.3–9.1)	0.762
Total cholesterol [mmol/L]	172.0 (157.0–203.5)	170.5 (161.5–199.0)	177.5 (151.0–219.0)	0.664
LDL [mmol/L]	93.5 (73.5–116.5)	93.5 (77.0–116.5)	92.5 (68.0–124.5)	0.985
HDL [mmol/L]	64.0 (54.0–70.0)	64.0 (53.5–69.5)	66.0 (54.5–71.0)	0.584
Triglycerides [mmol/L]	101.5 (87.0–114.5)	102.5 (84.0–112.5)	94.0 (88.0–128.0)	0.895
TG/HDL-c	1.67 (1.24–1.99)	1.52 (1.03–1.93)	1.77 (1.33–2.06)	0.458
WHR waist [cm]/hip [cm]	0.88 (0.83–0.92)	0.83 (0.79–0.87)	0.89 (0.86–0.94)	0.002
NDUFS8 protein [ng/mL]	2.25 (0.72–3.81)	2.90 (1.42–4.60)	1.22 (0.37–2.89)	0.008

**Table 2:** Univariate and multivariate regression analyses.

	OR (95% CI:). *p*	AOR (95% CI:). *p*
Age *	0.91 (0.85–0.98). 0.003	0.81 (0.67–0.97). 0.019
Diabetes duration *	0.93 (0.86–1.02). 0.105	0.99 (0.86–1.17). 0.002
Pack–years *	0.88 (0.69–1.11). 0.150	0.54 (0.25–1.18). 0.121
NDUFS8 protein *	1.71 (1.10–2.67). 0.005	2.38 (1.04–5.48). 0.042

Adjusted odds ratios (AOR) and confidence intervals (CI) were calculated in a multivariate logistic regression, and the *p*-value was calculated using the Wald test (*p* < 0.05 is assumed to be significant). The dependent variable was the median of eGDR (7.6 mg/kg/min). * continuous variable. Model significance level: *p* < 0.001.

**Table 3:** Multivariate logistic regression, dependent variable: serum concentration of NDUFS8 protein. R = 0.527; R^2^ = 0.278; F(4,31) = 2.9828; *p* < 0.03412; standard error of estimation: 1.8427.

N = 36	b *	Standard Error b *	b	Standard Error b	t (31)	*p*
Free word			−3.06635	1.952344	−1.57060	0.126
Age	0.281414	0.205495	0.04566	0.033343	1.36945	0.181
Duration of diabetes	−0.030173	0.189812	−0.00747	0.046986	−0.15896	0.875
Pack–years	−0.085595	0.158055	−0.00010	0.000176	−0.54155	0.592
e-GDR	0.554108	0.173041	0.54410	0.169915	3.20218	0.003

* continuous variable.

## Data Availability

The data presented in this study are available on request from the corresponding author. The data are not publicly available due to the presence of sensitive data.

## References

[1] Bluestone J.A., Herold K., Eisenbarth G. (2010). Genetics, pathogenesis and clinical interventions in type 1 diabetes. Nature.

[2] Giugliano D., Ceriello A., Paolisso G. (1996). Oxidative stress and diabetic vascular complications. Diabetes Care.

[3] Desai S., Deshmukh A. (2020). Mapping Of Type 1 Diabetes Mellitus. Curr. Diabetes Rev..

[4] Kretowski A., Kowalska I., Peczyñska J., Urban M., Green A., Kinalska I. (2001). The large increase in incidence of Type I diabetes mellitus in Poland. Diabetologia.

[5] Patterson C.C., Dahlquist G.G., Gyürüs E., Green A., Soltész G., Group E.S. (2009). Incidence trends for childhood type 1 diabetes in Europe during 1989–2003 and predicted new cases 2005-20: A multicentre prospective registration study. Lancet.

[6] Nielsen H.B., Ovesen L.L., Mortensen L.H., Lau C.J., Joensen L.E. (2016). Type 1 diabetes, quality of life, occupational status and education level—A comparative population-based study. Diabetes Res. Clin. Pract..

[7] Diabetes Control and Complications Trial Research Group (1994). Effect of intensive diabetes treatment on the development and progression of long-term complications in adolescents with insulin-dependent diabetes mellitus: Diabetes Control and Complications Trial. J. Pediatr..

[8] Šimonienė D., Platūkiene A., Prakapienė E., Radzevičienė L., Veličkiene D. (2020). Insulin Resistance in Type 1 Diabetes Mellitus and Its Association with Patient’s Micro- and Macrovascular Complications, Sex Hormones, and Other Clinical Data. Diabetes Ther..

[9] Mao Y., Zhong W. (2022). Changes of insulin resistance status and development of complications in type 1 diabetes mellitus: Analysis of DCCT/EDIC study. Diabetes Res. Clin. Pract..

[10] Lotfy M., Adeghate J., Kalasz H., Singh J., Adeghate E. (2017). Chronic Complications of Diabetes Mellitus: A Mini Review. Curr. Diabetes Rev..

[11] Cree-Green M., Stuppy J.J., Thurston J., Bergman B.C., Coe G.V., Baumgartner A.D., Bacon S., Scherzinger A., Pyle L., Nadeau K.J. (2018). Youth With Type 1 Diabetes Have Adipose, Hepatic, and Peripheral Insulin Resistance. J. Clin. Endocrinol. Metab..

[12] Priya G., Kalra S. (2018). A Review of Insulin Resistance in Type 1 Diabetes: Is There a Place for Adjunctive Metformin?. Diabetes Ther..

[13] Chillarón J.J., Goday A., Flores-Le-Roux J.A., Benaiges D., Carrera M.J., Puig J., Cano-Pérez J.F., Pedro-Botet J. (2009). Estimated glucose disposal rate in assessment of the metabolic syndrome and microvascular complications in patients with type 1 diabetes. J. Clin. Endocrinol. Metab..

[14] Zheng X., Huang B., Luo S., Yang D., Bao W., Li J., Yao B., Weng J., Yan J. (2017). A new model to estimate insulin resistance via clinical parameters in adults with type 1 diabetes. Diabetes Metab. Res. Rev..

[15] Uruska A., Araszkiewicz A., Zozulinska-Ziolkiewicz D., Uruski P., Wierusz-Wysocka B. (2010). Insulin resistance is associated with microangiopathy in type 1 diabetic patients treated with intensive insulin therapy from the onset of disease. Exp. Clin. Endocrinol. Diabetes.

[16] Chillarón J.J., Sales M.P., Flores-Le-Roux J.A., Murillo J., Benaiges D., Castells I., Goday A., Cano J.F., Pedro-Botet J. (2011). Insulin resistance and hypertension in patients with type 1 diabetes. J. Diabetes Complicat..

[17] Brownlee M. (2005). The pathobiology of diabetic complications: A unifying mechanism. Diabetes.

[18] Betteridge D.J. (2000). What is oxidative stress?. Metabolism.

[19] Cadenas E., Boveris A., Ragan C.I., Stoppani A.O. (1977). Production of superoxide radicals and hydrogen peroxide by NADH-ubiquinone reductase and ubiquinol-cytochrome c reductase from beef-heart mitochondria. Arch. Biochem. Biophys..

[20] Dröge W. (2002). Free radicals in the physiological control of cell function. Physiol. Rev..

[21] Cadenas E., Davies K.J. (2000). Mitochondrial free radical generation, oxidative stress, and aging. Free Radic. Biol. Med..

[22] Korshunov S.S., Skulachev V.P., Starkov A.A. (1997). High protonic potential actuates a mechanism of production of reactive oxygen species in mitochondria. FEBS Lett..

[23] Tang W.H., Martin K.A., Hwa J. (2012). Aldose reductase, oxidative stress, and diabetic mellitus. Front. Pharmacol..

[24] Wu J., Jin Z., Zheng H., Yan L.J. (2016). Sources and implications of NADH/NAD(+) redox imbalance in diabetes and its complications. Diabetes Metab. Syndr. Obes..

[25] Hernandez-Mijares A., Rocha M., Apostolova N., Borras C., Jover A., Bañuls C., Sola E., Victor V.M. (2011). Mitochondrial complex I impairment in leukocytes from type 2 diabetic patients. Free Radic. Biol. Med..

[26] Victor V.M., Rocha M., Bañuls C., Sanchez-Serrano M., Sola E., Gomez M., Hernandez-Mijares A. (2009). Mitochondrial complex I impairment in leukocytes from polycystic ovary syndrome patients with insulin resistance. J. Clin. Endocrinol. Metab..

[27] Koopman W.J., Verkaart S., Visch H.J., van Emst-de Vries S., Nijtmans L.G., Smeitink J.A., Willems P.H. (2007). Human NADH:ubiquinone oxidoreductase deficiency: Radical changes in mitochondrial morphology?. Am. J. Physiol. Cell Physiol..

[28] De Sury R., Martinez P., Procaccio V., Lunardi J., Issartel J.P. (1998). Genomic structure of the human NDUFS8 gene coding for the iron-sulfur TYKY subunit of the mitochondrial NADH:ubiquinone oxidoreductase. Gene.

[29] Procaccio V., Wallace D.C. (2004). Late-onset Leigh syndrome in a patient with mitochondrial complex I NDUFS8 mutations. Neurology.

[30] DeFronzo R.A., Tobin J.D., Andres R. (1979). Glucose clamp technique: A method for quantifying insulin secretion and resistance. Am. J. Physiol..

[31] Akbari M., Kirkwood T.B.L., Bohr V.A. (2019). Mitochondria in the signaling pathways that control longevity and health span. Ageing Res. Rev..

[32] Song X., Hu W., Yu H., Wang H., Zhao Y., Korngold R. (2020). Existence of Circulating Mitochondria in Human and Animal Peripheral Blood. Int. J. Mol. Sci..

[33] Torralba D., Baixauli F., Sánchez-Madrid F. (2016). Mitochondria Know No Boundaries: Mechanisms and Functions of Intercellular Mitochondrial Transfer. Front. Cell Dev. Biol..

[34] Puhm F., Afonyushkin T., Resch U., Obermayer G., Rohde M., Penz T., Schuster M., Wagner G., Rendeiro A.F., Melki I. (2019). Mitochondria Are a Subset of Extracellular Vesicles Released by Activated Monocytes and Induce Type I IFN and TNF Responses in Endothelial Cells. Circ. Res..

[35] Pollara J., Edwards R.W., Lin L., Bendersky V.A., Brennan T.V. (2018). Circulating mitochondria in deceased organ donors are associated with immune activation and early allograft dysfunction. JCI Insight.

[36] Phinney D.G., Di Giuseppe M., Njah J., Sala E., Shiva S., St Croix C.M., Stolz D.B., Watkins S.C., Di Y.P., Leikauf G.D. (2015). Mesenchymal stem cells use extracellular vesicles to outsource mitophagy and shuttle microRNAs. Nat. Commun..

[37] Al Amir Dache Z., Otandault A., Tanos R., Pastor B., Meddeb R., Sanchez C., Arena G., Lasorsa L., Bennett A., Grange T. (2020). Blood contains circulating cell-free respiratory competent mitochondria. FASEB J..

[38] Stier A. (2021). Human blood contains circulating cell-free mitochondria, but are they really functional?. Am. J. Physiol. Endocrinol. Metab..

[39] Mahrouf-Yorgov M., Augeul L., Da Silva C.C., Jourdan M., Rigolet M., Manin S., Ferrera R., Ovize M., Henry A., Guguin A. (2017). Mesenchymal stem cells sense mitochondria released from damaged cells as danger signals to activate their rescue properties. Cell Death Differ..

[40] Zhao Y., Jiang Z., Delgado E., Li H., Zhou H., Hu W., Perez-Basterrechea M., Janostakova A., Tan Q., Wang J. (2017). Platelet-Derived Mitochondria Display Embryonic Stem Cell Markers and Improve Pancreatic Islet β-cell Function in Humans. Stem Cells Transl. Med..

[41] Zhao Y., Huang Z., Lazzarini P., Wang Y., Di A., Chen M. (2007). A unique human blood-derived cell population displays high potential for producing insulin. Biochem. Biophys. Res. Commun..

[42] Grazioli S., Pugin J. (2018). Mitochondrial Damage-Associated Molecular Patterns: From Inflammatory Signaling to Human Diseases. Front. Immunol..

[43] Rodríguez-Nuevo A., Zorzano A. (2019). The sensing of mitochondrial DAMPs by non-immune cells. Cell Stress.

[44] Herzig S., Shaw R.J. (2018). AMPK: Guardian of metabolism and mitochondrial homeostasis. Nat. Rev. Mol. Cell Biol..

[45] Burkewitz K., Zhang Y., Mair W.B. (2014). AMPK at the nexus of energetics and aging. Cell Metab..

[46] Hardie D.G., Ross F.A., Hawley S.A. (2012). AMPK: A nutrient and energy sensor that maintains energy homeostasis. Nat. Rev. Mol. Cell Biol..

[47] Egan D.F., Shackelford D.B., Mihaylova M.M., Gelino S., Kohnz R.A., Mair W., Vasquez D.S., Joshi A., Gwinn D.M., Taylor R. (2011). Phosphorylation of ULK1 (hATG1) by AMP-activated protein kinase connects energy sensing to mitophagy. Science.

[48] Pollard A.K., Craig E.L., Chakrabarti L. (2016). Mitochondrial Complex 1 Activity Measured by Spectrophotometry Is Reduced across All Brain Regions in Ageing and More Specifically in Neurodegeneration. PLoS ONE.

[49] Petrosillo G., Matera M., Moro N., Ruggiero F.M., Paradies G. (2009). Mitochondrial complex I dysfunction in rat heart with aging: Critical role of reactive oxygen species and cardiolipin. Free Radic. Biol. Med..

[50] Zhao Y., Wang H., Mazzone T. (2006). Identification of stem cells from human umbilical cord blood with embryonic and hematopoietic characteristics. Exp. Cell Res..

[51] Zhao Y., Jiang Z., Zhao T., Ye M., Hu C., Yin Z., Li H., Zhang Y., Diao Y., Li Y. (2012). Reversal of type 1 diabetes via islet β cell regeneration following immune modulation by cord blood-derived multipotent stem cells. BMC Med..

[52] Li Y., Yan B., Wang H., Li H., Li Q., Zhao D., Chen Y., Zhang Y., Li W., Zhang J. (2015). Hair regrowth in alopecia areata patients following Stem Cell Educator therapy. BMC Med..

[53] Delgado E., Perez-Basterrechea M., Suarez-Alvarez B., Zhou H., Revuelta E.M., Garcia-Gala J.M., Perez S., Alvarez-Viejo M., Menendez E., Lopez-Larrea C. (2015). Modulation of Autoimmune T-Cell Memory by Stem Cell Educator Therapy: Phase 1/2 Clinical Trial. EBioMedicine.

[54] Zhao Y., Jiang Z., Zhao T., Ye M., Hu C., Zhou H., Yin Z., Chen Y., Zhang Y., Wang S. (2013). Targeting insulin resistance in type 2 diabetes via immune modulation of cord blood-derived multipotent stem cells (CB-SCs) in stem cell educator therapy: Phase I/II clinical trial. BMC Med..

[55] Chen J., Stimpson S.E., Fernandez-Bueno G.A., Mathews C.E. (2018). Mitochondrial Reactive Oxygen Species and Type 1 Diabetes. Antioxid. Redox Signal..

[56] Matulewicz N., Karczewska-Kupczewska M. (2016). Insulin resistance and chronic inflammation. Postepy Hig. I Med. Dosw..

[57] Park K., Gross M., Lee D.H., Holvoet P., Himes J.H., Shikany J.M., Jacobs D.R. (2009). Oxidative stress and insulin resistance: The coronary artery risk development in young adults study. Diabetes Care.

[58] Mijaljica D., Prescott M., Devenish R.J. (2010). Mitophagy and mitoptosis in disease processes. Methods Mol. Biol..

[59] Gerber P.A., Rutter G.A. (2017). The Role of Oxidative Stress and Hypoxia in Pancreatic Beta-Cell Dysfunction in Diabetes Mellitus. Antioxid. Redox Signal..

[60] Hurrle S., Hsu W.H. (2017). The etiology of oxidative stress in insulin resistance. Biomed. J..

[61] Chen J., Gusdon A.M., Mathews C.E. (2011). Role of genetics in resistance to type 1 diabetes. Diabetes Metab. Res. Rev..

[62] Dionne F.T., Turcotte L., Thibault M.C., Boulay M.R., Skinner J.S., Bouchard C. (1993). Mitochondrial DNA sequence polymorphism, VO2max, and response to endurance training. Med. Sci. Sports Exerc..

